# Evaluation of Syncope: An Overview

**Published:** 2001-10-01

**Authors:** Anoop Kumar Gupta, Alok Maheshwari, Yash Lokhandwala

**Affiliations:** Michigan State University, Lansing, MI, USA

## Introduction

Syncope is defined as a sudden temporary loss of consciousness associated with a loss of postural tone, with spontaneous recovery that does not require electrical or chemical cardioversion. Syncope is a common symptom, accounting for 1% to 6% of hospital admission and up to 3% of emergency room visits. Loss of consciousness is also common in healthy young adults, although most do not seek medical attention. Syncope is a frequent symptom in the elderly.

The evaluation and management of syncope has dramatically changed over the past 15 years. In the early 1980s, several studies showed that the cause of syncope was often not established, and subgroups were identified with high mortality and sudden death rates [1-4].  Later a large number of studies on electrophysiology testing appeared, which led to a better understanding of the roles and limitations of tests in syncope [5-8].  Although tilt table testing started in 1980s, it assumed an important role in the evaluation of syncope in 1990s, showing that neurally mediated mechanism is a common etiology of unexplained syncope [9-12]. 

The purpose of this article is to highlight the clinical approach and management of syncope.

## Approach to the patient with syncope

The proper diagnostic and therapeutic approach requires careful analysis of the patient's symptoms and of the clinical findings. No specific battery of tests is ever indicated or is always useful. Extensive diagnostic evaluation is generally unnecessary, expensive, and risky. Repeated evaluation and hospital admission after an initial complete negative assessment is often unrewarding.

Since it is clearly impractical to wait to monitor all episodes of syncope in order to arrive at a diagnosis with the present technology, clinicians must base their decisions on historical features with the presumption that the description of the episode is accurate, complete, and based on common sense [13-15].  The proper evaluation requires a balance of the judicious use of inpatient and outpatient diagnostic modalities. The expense and risk of the procedures and of hospitalization are intensified by the possibility of iatrogenic harm caused by diagnostic or therapeutic misadventures.

## The History

To evaluate syncope, sound clinical decisions are based on a carefully performed history with extreme attention to detail. The history, with its proper interpretation (Table 1) and a directed physical examination, is the only appropriate way to guide further diagnostic evaluation. The history and physical alone can be diagnostic in 25%-35% of patients [1,4,16]. Of those for whom a cause is found, the history and physical alone are sufficient in 75%-85% of patients [4]. Specific attention should be directed toward: 1) characteristic and length of the episode, 2) patient and  witnessed accounts, 3) patient age, 4) concomitant (specially cardiac) disease, 5) associated temporally related symptoms (e.g., neurological symptoms, angina, palpitations, and heart failure),6) premonitory (prodromal) symptoms, 7) symptoms on awakening (post syncope symptoms), 8) the circumstances, situations surrounding the episode, 9) exercise, body position, posture, and emotional state, 10) number, frequency, and timing of previous syncopal episodes, 11) medications, and 12) family history. As part of the initial assessment, early determination of the presence of heart disease is especially crucial because these patients are at the highest risk for death.

## Physical Examination

The physical examination can provide important clues to support a diagnosis suspected from the patient's history. Attention should be directed to the vital signs, the cardiovascular examination, and neurological examination (Table 2 ).

### Vital signs

This includes blood pressures in supine, sitting, and standing, initially and after several minutes, with attention to change in the heart rate and symptoms. An abrupt drop in blood pressure with standing, especially with reproduction of symptoms, suggests volume depletion as a potential cause. The heart rate should rise with standing in a volume-depleted patient. In patients with idiopathic orthostatic hypotension, diabetes, amylodosis, and autonomic insufficiency, the blood pressure can drop over several minutes in the standing position and the heart rate may not change.

An evaluation of the pulse can provide insight into the presence of a dissecting aneurysm or subclavian steal. The carotid impulse may reveal evidence for aortic stenosis but a carotid bruit does not provide a direct cause of syncope. It may indicate, however, the presence of other atherosclerotic lesions such as coronary artery disease (cardiac cause of syncope) or subclavian artery occlusion (subclavian steal-related syncope). Carotid sinus massage can give insight into carotid sinus hypersensitivity, which is more common in elderly. There should be no bruit on auscultation of the carotid before the massage is performed.

### Cardiovascular Examination

This may reveal murmurs consistent with hypertrophic cardiomyopathy, aortic stenosis, mitral valve prolapse, tricuspid regurgitation, or pulmonary hypertension. Valsalva maneuver can diagnose hypertrophic cardiomyopathy clinically.   The presence of S4 and S3 gallops, are potential indicators of cardiac disease, which may be responsible for syncope. An S3 gallop could indicate the presence of congestive heart failure.  Evidence of Eisenmenger's syndrome, pulmonic stenosis, prosthetic valve dysfunction, aortic stenosis, or a tumor flop can provide further clues for the diagnosis of syncope.

### Neurological assessment

The neurological evaluation may indicate focal or localizing signs or evidence for a systemic neurological signs such as Parkinson's disease. Changing neurological signs are also important. A new neurological deficit in a patient with syncope should be considered a premonitory sign for a cerebrovascular accident.

## Diagnostic testing

The proper diagnostic approach requires careful analysis of syncope in light of all available clinical findings. When used properly, they will increase the diagnostic yield compared to the history and physical alone. All testing must be tailored to the patient based on the findings of the history and physical examinations and with knowledge of the sensitivity and specificity of each test to identify the cause of syncope.

### Baseline laboratory tests

Initial laboratory blood tests are generally not abnormal and they generally do not lead to a diagnosis. Hypoglycemia, hyponatremia, hypocalcemia, or renal failure is found in 2% to 3% of patients, but these appear to be patients with seizures rather than syncope [1-4].  These abnormalities are often suspected clinically. Bleeding is generally diagnosed clinically and confirmed by a complete blood count or hemoccult tests.

### Cardiovascular testing

#### 12-lead ECG

Although ECG is often abnormal, causes of syncope are rarely assigned (<5% of patients) on the basis of ECG and rhythm strip [1-4].  An ECG is recommended in all patients with syncope because abnormalities found on ECG (such as bundle branch block) may guide further evaluation, or if a specific diagnosis is made the findings can be important in immediate decision making.

#### Prolonged ECG monitoring

It has become clear that results of ambulatory monitoring are often difficult to interpret in evaluating syncope because of the lack of a "gold standard" for diagnosis of arrhythmias and the rarity of symptoms during monitoring. The best way to assess the usefulness of ambulatory monitoring is to use presence or absence of symptoms during monitoring [17]. Approximately 4% of patients have symptoms concurrently with arrhythmias, and 17% have symptoms but no arrhythmias, thus potentially excluding arrhythmias as a cause of symptoms. In approximately 79% of patients there are no symptoms, but brief arrhythmias are found in 13%. In the absence of symptoms during monitoring, finding brief or no arrhythmias does not exclude arrhythmic syncope. Brief arrhythmias are nonspecific and can be found in asymptomatic healthy individuals. Additionally, absence of arrhythmias on monitoring does not exclude arrhythmic syncope because arrhythmias are episodic and may not be captured during monitoring. In patients with high pretest probability of arrhythmias such as brief sudden loss of consciousness without prodrome, patients with abnormal ECG, or those with structural heart disease, arrhythmias are still of concern and further testing is needed. Holter monitoring for 72 hours rather than for 24 hours does not yield greater numbers of symptomatic periods [18].

#### Long-term ambulatory loop event monitoring

Loop event monitor can be activated after a syncopal episode, and can record 2 to 5 minutes of rhythm strips prior to the activation and 30 to 60 seconds of the rhythm after the activation. Tracings can be transmitted via telephone and monitors can be worn for weeks to months. Studies of loop monitoring show that arrhythmias with symptoms are found in 8% to 20% of patients. In additional 27%, there are symptoms without concurrent arrhythmias [19].  This test is recommended in patients with recurrent event during the monitoring period.

#### Electrophysiological studies

In patients with structural heart disease and/or abnormal ECG, the diagnostic yield of EPS is approximately 50%, whereas it is only 10% in patients without structural heart disease [5-8].  Bradyarrhythmias are much more likely to be diagnosed in patients with conduction disease on surface ECG, however, the sensitivity and specificity of EPS for detection of bradyarrhythmias is low.

It is recommended that patients with structural heart disease or abnormal ECG undergo electrophysiological testing if clinical assessment is suggestive of arrhythmic syncope and if noninvasive testing with Holter or loop monitoring has been non-diagnostic.

#### Signal-Averaged ECG

Finding low-amplitude signals (late potentials) has a sensitivity of 73% to 89% and specificity of 89% to 100% for prediction of inducible sustained ventricular tachycardia by EPS [20-21]. This test may be useful in deciding if there is a need for electrophysiological studies for diagnosis of ventricular tachycardia when these arrhythmias are the only concern. However, EPS is often performed for diagnosis of tachyarrhythmias. This test is not likely to be useful under such circumstances because complete assessment is needed.

#### Carotid Massage

In the absence of symptoms reproduction, carotid sinus syncope is likely when carotid sinus hypersensitivity is found and either 1) spontaneous episode are related to activities that press or stretch the carotid sinus or 2) patient has recurrent syncope with a negative work-up.

Survival of patients with carotid sinus hypersensitivity is similar to that of general population and largely related to underlying disease. Survival appears to be unrelated to pacemaker therapy [22]. Symptoms recur in 20% to 25% of untreated or medically treated patients with carotid sinus syndrome.

Carotid massage is recommended when symptoms are suggestive of carotid sinus syncope and in elderly patients with unexplained syncope. Although carotid sinus massage is usually performed in the supine position, performing massage during head-up tilt testing may increase the diagnostic yield. This was illustrated by one study of 80 patients with unexplained syncope, 30 controls, and 16 patients with syncope not related to carotid sinus hypersensitivity [23]. Carotid sinus hypersensitivity was elicited by carotid sinus massage in the supine position in 8.7 percent of those with unexplained syncope; when repeated during tilt table testing, carotid hypersensitivity was observed in 60 percent. Among controls and those with syncope of other causes, the incidence of carotid hypersensitivity was similar with and without the tilt table test (6.6 and 6.3 percent, respectively).

#### Echocardiogram

Echocardiography in the absence of clinical evidence of organic heart disease generally does not reveal unexpected findings that lead to an etiology for syncope [24].  This test is not recommended for screening purpose in patients with syncope.

#### Exercise testing

The yield of exercise testing in the diagnosis of the etiology of syncope is very low (<1%). Exercise testing is useful as an ancillary diagnostic test for evaluation of ischemic heart disease in patients with arrhythmic syncope, particularly ventricular tachycardia. In these patients, in addition to the treatment of ventricular tachyarrhythmia, the management of underlying cardiac disease is critical. Exercise ECG is also recommended for the evaluation of symptoms with exercise and post exertional syncope.

#### Upright Tilt Testing

The tilt table test (also called the upright tilt table test) has become a commonly performed test for the evaluation of syncope, particularly in young, otherwise healthy patients in whom the diagnosis of vasovagal or neurocardiogenic syncope is often entertained [11,25].  It is also useful in older persons with suspected neurally mediated syncope [26].  Maintaining the patient in an upright position for a brief duration on a tilt table has become a common means of testing for predisposition to vasovagal syncope. It is widely accepted that hypotension and /or bradycardia during upright tilt testing is equivalent to spontaneous vasovagal syncope. This is supported by the fact that the temporal sequence of blood pressure and heart rate changes during tilt testing is similar to spontaneous spells. In addition, catecholamine release immediately prior to tilt-induced syncope is similar to spontaneous vasovagal faint.

Two general types of testing procedures include upright tilt testing alone (passive testing) and tilt testing in conjunction with a chemical agent [9-12].  A vast majority of the reported studies employ passive testing or use isoproterenol after a brief period of passive tilt testing.

There are various positive response patterns seen during head-up tilt table testing. 1) Classical vasovagal (or neurocardiogenic), which is characterized by the sudden onset of hypotension with or without coexistent bradycardia. Vasovagal response can be cardioinhibitory, pure vasodepressor, or mixed type. 2) dysautonomic response, characterized by gradual parallel decline in systolic and diastolic blood pressure, leading to loss of consciousness.3) psychogenic or psychosomatic response, these patients experience syncope during tilt testing with no ascertainable alteration in heart rate, blood pressure, electroencephalographic, or transcranial blood flow patterns.4) Postural Orthostatic Tachycardia Syndrome (POTS), characterized by an increase in heart rate of at least 30 beats per minute (or a maximum heart rate of 120 bpm) within the first 10 minutes upright during the baseline tilt, this tachycardia is not associated with profound hypotension.

##### Response to tilt table test

The test is usually performed in an electrophysiology laboratory using a special tilt table; isoproterenol is often infused if the initial tilt test is negative [27]. Occasional patients have a pronounced cardioinhibitory response to this test characterized by symptomatic hypotension, bradycardia, or both . In one series, 77 of 179 patients (43 percent) with unexplained syncope had a positive tilt test; 10 of these patients developed asystole, often associated with seizures, requiring a brief period of cardiopulmonary resuscitation [28].

The false negative rate of the upright tilt table test is as high as 14 percent and up to 30 percent when isoproterenol is infused [29,30] and is less specific in the elderly [31]. The mechanism for syncope during tilt table testing is different in normal and patients with a history of neurocardiogenic syncope. One study compared 8 normal with and 8 normal without a positive tilt table test and 15 patients with neurocardiogenic syncope [32]. Patients with neurocardiogenic syncope had a shorter time to syncope than normal subjects with a false positive study; an immediate and persistent drop in mean blood pressure, suggesting impaired vascular resistance response; more rapid peripheral pooling of blood, as determined by left ventricular end-diastolic dimension on echocardiography; and higher peak epinephrine levels.

Tilt table testing should not be performed in a patient who is orthostatic at baseline or who has had near-syncope without overt loss of consciousness. The utility of the tilt table test depends upon the study population. One report evaluated the role of this test in 145 patients with a history of presyncope or syncope. The following findings were noted [33]:

- Patients with recurrent syncope were more likely to have a positive test compared to those with a single episode or with recurrent presyncope (41 versus 17 percent, p<0.005).
- Patients with structural heart disease or with a noncardiovascular cause for syncope were less likely to have a positive test (16 versus 42 percent, p<0.0001).
- When multiple factors were combined, the yield ranged from 0 percent in patients under 50 without recurrent syncope who had structural heart disease or a noncardiovascular cause to 73 percent in those over 50 with recurrent syncope who did not have structural heart disease or a non-cardiovascular cause.
- The additional yield of positive tests with the use of isoproterenol or edrophonium was 10 percent.

Another study evaluated the role of the tilt study in patients with bifascicular block who had unexplained syncope. When compared to those with bifascicular block and no syncope, no difference in the incidence of a positive tilt test was observed, suggesting that test specificity in this population is of concern [34]. Tilt testing is also of limited value for patients with situational vagal syncope, i.e., syncope due to situations associated with enhanced vagal tone such as micturition, defecation, cough, or vomiting [35].

##### Protocol for tilt table testing

The basic facilities and equipment necessary for performing a tilt table test are straightforward. The test should be performed in a quiet room with minimal distractions for the patient. The patient should be placed on a hydraulic lift or swinging bed capable of moving the patient passively from a supine position to a head-up position between 60° and 90°. The table must also have a footboard and safety restraints. Continuous ECG and noninvasive blood pressure monitoring are employed throughout the test. Patients should be encouraged to help identify symptoms that may develop. An infusion pump and intravenous catheter are necessary for administration of fluids and isoproterenol if indicated. Patients should be in a semi fasting state without orthostatic blood pressure changes at baseline.

A variety of protocols have been described for the test that vary in the angle of tilt (60° to 90°), duration of tilt (10 to 60 minutes), and the administration of isoproterenol. In general, the patient is monitored in the supine position for 5 minutes to obtain baseline heart rate and blood pressure measurements. The patient is then positioned in a head-up tilt position. Blood pressure, heart rate, and symptoms are recorded every 3 to 5 minutes and the ECG is recorded continuously. If the patient experiences loss of consciousness or is unable to maintain posture in association with a significant fall in blood pressure or heart rate, he or she is returned to a supine position, and the test is considered positive. If, after a period of 10 to 60 minutes, no symptoms have developed, the patient is returned to the supine position.

If the patient has remained asymptomatic, the majority of investigators will perform a second tilt while infusing isoproterenol [27]. One study found that a single-stage isoproterenol tilt table test more frequently induced syncope than a standard passive tilt study (56 versus 32 percent) and reduced the time necessary for the procedure. There was, however, a lower specificity (83 versus 91 percent for standard tilt testing) [36]. Although isoproterenol increases the frequency of positive tests, it also increases the number of false positive results and decreases the specificity of the tilt table test. The infusion of isoproterenol is usually titrated to achieve a 20 to 30 percent increase in the baseline heart rate while the patient is supine. The patient is then placed in the head-up tilt position for an additional 20 to 30 minutes.

Many investigators require either loss of consciousness or postural tone to consider the test positive while isoproterenol is infused. A modest decrease in blood pressure with symptoms is common with isoproterenol and nonspecific.

Although a controlled infusion of isoproterenol is safe in patients without heart disease, it should not be used in those with coronary artery disease since angina and serious arrhythmia can be provoked [37,38].

In order to reduce the time necessary for the tilt test, one study of 109 patients reported that a heart rate change ≤18 beats per minute during the first six minutes of the test prospectively predicted a negative tilt table study with a 96.4 percent specificity, 98.4 percent positive predictive accuracy and 87.3 percent sensitivity, even with the subsequent use of isoproterenol [39].

##### Adenosine and nitrates

Adenosine may have a complementary role to the tilt table test in the evaluation of patients with possible vasovagal syncope, since it may increase sympathetic discharge by activation of cardiovascular afferent nerves; importantly, the use of adenosine requires less time, often less than 10 minutes. One study, for example, evaluated the utility of adenosine (given as 6 and 12 mg boluses) compared to head-up tilt-table testing in 85 patients presenting with syncope and in 14 normal controls [40]. A vasovagal response was defined as the development of syncope or presyncope associated with relative bradycardia and/or hypotension (decrease in systolic blood pressure  ≥ 30 mmHg) occurring 15 to 60 sec after adenosine injection or during the tilt table test. The inducibility of a vasovagal response with adenosine was comparable to that with the tilt table test in patients with syncope (26 and 34 percent) and in normal (7 percent for both tests). These observations also suggest that adenosine may be an endogenous modulator of the vasovagal response [41].

However, a second study of 100 patients found that, despite a similar yield, results with adenosine and routine tilt table study were discordant in 21 percent of patients; however, most of the patients with a positive response to adenosine but negative tilt table study had a positive response with the use of isoproterenol, suggesting that adenosine and isoproterenol tilt testing have complementary roles [41].

Another pharmacological agent that may have a complementary role to the tilt table study is sublingual or intravenous nitrates [42-44]. In one report, the use of sublingual isosorbide dinitrate increased the frequency of a positive tilt study among patients with a history of vasovagal syncope from 13 to 87 percent, although the number of normal with a positive tilt study also increased from 0 to 6 percent [42]. One study compared sublingual nitroglycerin to isoproterenol in 71 patients with unexplained syncope and 30 controls and found that the diagnostic accuracy was similar; however, sublingual nitroglycerin was simpler to use, better tolerated, and safer than low-dose isoproterenol [45].

##### Clomipramine

Acute clomipramine administration blocks the reuptake of serotonin in the synapse space and increases stimulation of serotonin receptors and responsiveness of the central serotoninergic nervous system in subjects with vasovagal syncope; this leads to sympathetic withdrawal. One study of 55 patients with a history of neurocardiogenic syncope found that an intravenous infusion of clomipramine (5 mg in 5 min) increased the number of patients with a positive tilt table test (80 versus 53 percent without clomipramine); only 1 of 22 controls had a positive test with the drug [46].

Upright tilt testing is recommended for patients with recurrent  unexplained syncope in whom cardiac causes have been excluded or are not likely. In patients with negative passive tests and a high likelihood of neurally mediated syncope clinically (e.g., young person with concurrent autonomic symptoms), additional testing with isoproterenol is recommended.

#### Neurological testing

Generally, skull films, lumbar puncture, radionuclide brain scan, carotid Doppler's, and cerebral angiography do not yield diagnostic information for a cause of syncope in the absence of clinical findings that are suggestive of a specific neurological process [1].  Studies of EEG in syncope have shown that an epileptiform abnormality was found in 1% of patients; almost all of these were suspected clinically [47]. Head CT scans are rarely useful to assign an etiology, but are needed if subdural bleed due to head injury is suspected or in patients suspected to have seizures as a cause of loss of consciousness [1].

#### Psychiatric Assessment

Psychiatric illnesses must be considered as a cause of syncope, especially in young patients and those with multiple syncopal episodes who also have other nonspecific symptoms [48]. The disorders that may cause syncope include generalized anxiety and panic disorders, major depression, somatization disorder, and alcohol/substance abuse. Screening instruments for these disorders are available and recommended.

## Summary and conclusion

Syncope is a common manifestation of many disease processes. In a minority of cases, the problem is recurrent and handicapping. Patient with syncope and heart disease, particularly when there is impaired left ventricular function, bundle branch block, evidence of congestive heart failure, or a positive family history of syncope and heart disease, appear to be at high risk for death and require an aggressive initial approach. Patients who benefit most from hospitalization include those with suspected cardiac disease, the elderly, those with serious injuries, and those with new neurological findings.

Diagnostic test should be used sparingly, directed by a carefully performed history and physical examination. No series of test is universally applicable.

## Figures and Tables

**Figure 1:**
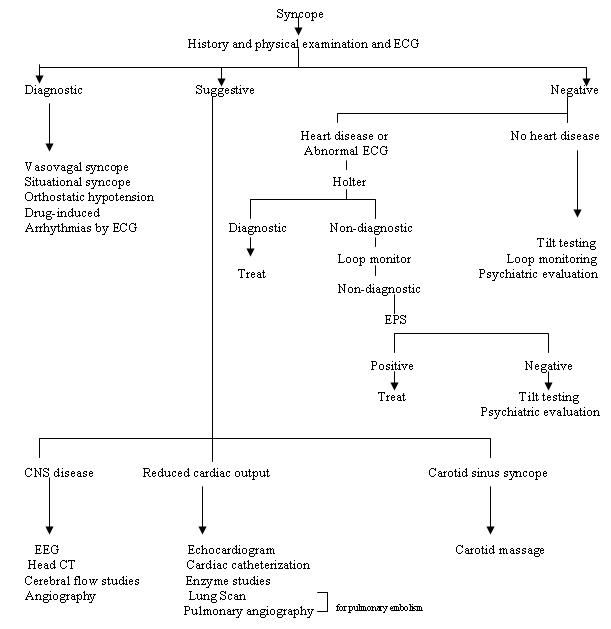
Approach to syncope

**Table 1:**
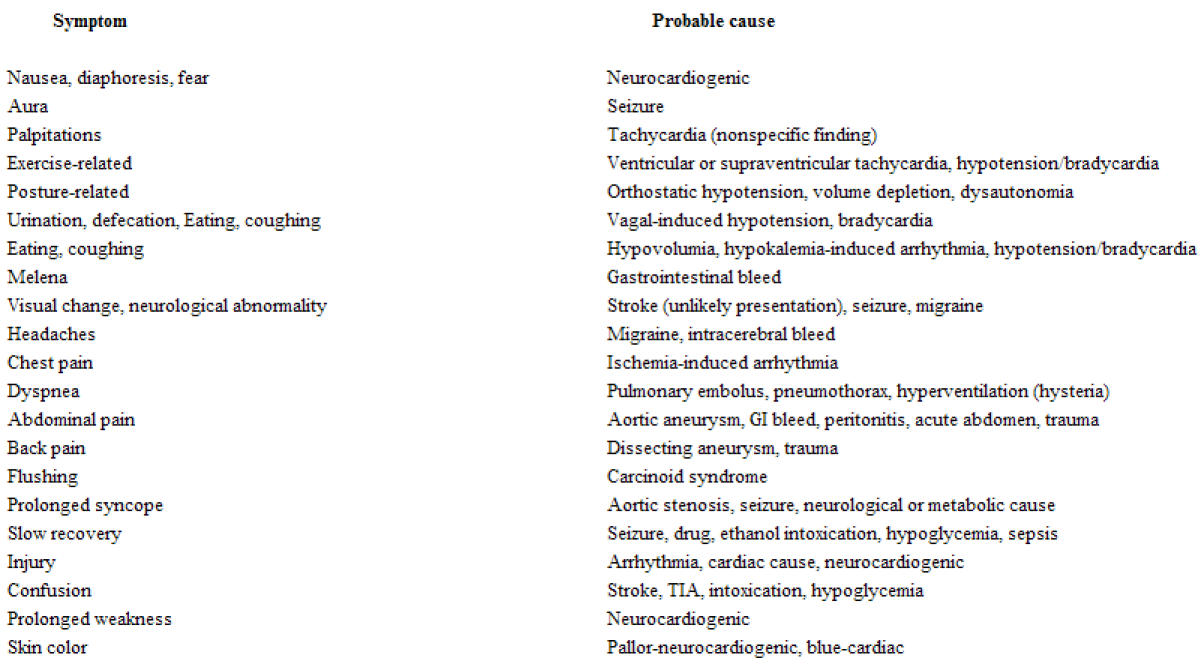
History: Symptoms related to syncopal spell

**Table 2:**
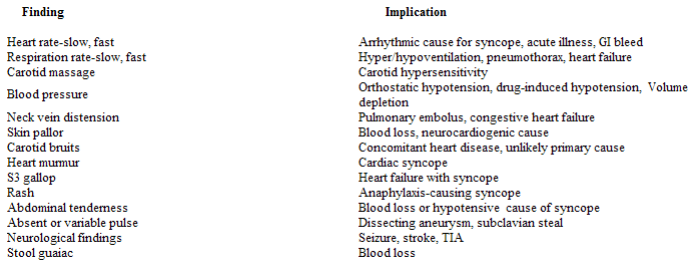
Physical findings: Key points
